# Decreased serum 4-Hydroxynonenal level as a biomarker for the progression of steroid-induced osteonecrosis of the femoral head

**DOI:** 10.1186/s13018-023-04153-1

**Published:** 2023-09-27

**Authors:** Fangjun Xiao, Tengfei Wei, Huan Xiao, Wei He, Qiushi Wei

**Affiliations:** 1 Guangzhou University of Chinese Medicine, Guangzhou, China; 2 Department of Orthopedics, Bijie Hospital of Chinese Medicine, Bijie, Guizhou China; 3 Guangdong Research Institute for Orthopedics and Traumatology of Chinese Medicine, Guangzhou, China; 4 Department of Orthopaedics, The Third Affiliated Hospital of Guangzhou University of Chinese Medicine, Guangzhou, China; 5 Lingnan Medical Research Center of Guangzhou University of Chinese Medicine, Guangzhou, China

**Keywords:** Oxidative stress, 4-Hydroxynonenal, Steroid-induced osteonecrosis of the femoral head, Biomarker, Collapse

## Abstract

**Background:**

Osteonecrosis of the femoral head (ONFH) is becoming a prevalent global health problem. 4-Hydroxynonenal (4-HNE) serves as a common marker of oxidative stress. This study aims to study the potential role of 4-HNE in the progression of steroid-induced osteonecrosis of the femoral head (SIONFH).

**Method:**

Between April 2021 and December 2021, 64 subjects were enrolled in this cross-sectional case‒control study. Thirty-six patients were grouped based on the Association Research Circulation Osseous (ARCO) classification, and 28 healthy volunteers without hip pain or any lesions shown in anteroposterior and frog-leg lateral pelvic radiographs served as the normal control group. Bone hematoxylin–eosin (HE) staining, microcomputed tomography (micro-CT), immunohistochemistry, and levels of plasma 4-HNE were evaluated.

**Results:**

The 4-HNE level was higher in the SIONFH group than in the normal control group (*P* < 0.001), and 4-HNE levels were significantly higher in SIONFH patients in the early stage of disease (stage II). The 4-HNE level was negatively correlated with ARCO stage (*r* = − 0.6875, *P* < 0.001). Immunohistochemistry revealed the presence of 4-HNE in the trabecular bone, osteocytes, and bone marrow.

**Conclusion:**

The 4-HNE level is negatively associated with ARCO stages. Lower levels of 4-HNE may serve as a critical biomarker for the progression of SIONFH.

## Introduction

Osteonecrosis of the femoral head (ONFH) is a devastating disease, and femoral head structural changes or even femoral head collapse and hip joint destruction are the main features of this disease [1]. The early stages of ONFH are frequently asymptomatic but may also present with hip pain and limited joint range of motion on physical examination [2, 3]. Early diagnosis of ONFH provides physicians with treatment options beyond total hip replacement (THA) [4]. Without early intervention, 70–80% of ONFH patients will develop secondary hip arthritis, necessitating THA [5–8]. The prevalence of ONFH is on the rise, making it a growing global health concern [9, 10]. The aetiology of ONFH includes both traumatic and nontraumatic causes. Nontraumatic ONFH is associated with many risk factors, including steroid use, alcohol consumption, and autoimmune diseases, such as systemic lupus erythematosus and rheumatoid arthritis [11–13]. In particular, steroid-induced ONFH (SIONFH) occurs frequently among young and middle-aged individuals [14]. Previous studies have found that high-dose corticosteroid use (> 20 mg prednisone equivalents per day) is related to ONFH [2, 15]. Developing highly reliable methods for preventing SIONFH is imperative.

The exact mechanisms underlying SIONFH remain unclear, but it is widely accepted that the basic mechanism of SIONFH involves circulation interruption to a specific area, leading to necrosis [1]. Researchers have proposed various intravascular mechanisms for the associated ischaemic process, including oxidative stress, blood thrombosis or coagulation, lipid metabolism abnormalities, and cell apoptosis [16]. Oxidative stress arises from an imbalance between reactive oxygen species (ROS) production and the ability of a biological system to readily detoxify reactive intermediates or easily repair damage [17]. When additional oxidative events occur, the pro-oxidant systems outbalance the antioxidants, potentially producing oxidative damage to lipids, proteins, carbohydrates, and nucleic acids, ultimately leading to cell death [18]. Some studies have suggested that suppressing oxidative stress may prevent the occurrence and progression of SIONFH [19, 20]. Thus, this research focuses on the role of oxidative stress in the progression of SIONFH.

4-Hydroxynonenal (4-HNE) is one of the key bioactive products of polyunsaturated fatty acid peroxidation. Due to their high affinity for proteins, 4-HNE protein adducts have been recognized as reliable and stable biomarkers for oxidative stress-related diseases [21, 22]. Previous studies have extensively investigated the role of 4-HNE in neurodegenerative diseases [23], diabetes [24], heart disease [25], atherosclerosis [26], and cancers [27]. Additionally, some studies have indicated that 4-HNE acts as a growth-regulating factor that may interfere with the activity of cytokines and influence the growth of cultured human bone cells [28]. However, the possible involvement of 4-HNE in the collapse of SIONFH remains unexplored. Therefore, this study aims to compare the plasma levels of 4-HNE in patients with SIONFH at various stages, evaluating its potential as a blood-based biomarker for SIONFH.

## Materials and methods

### Study population

**Fig. 1:**
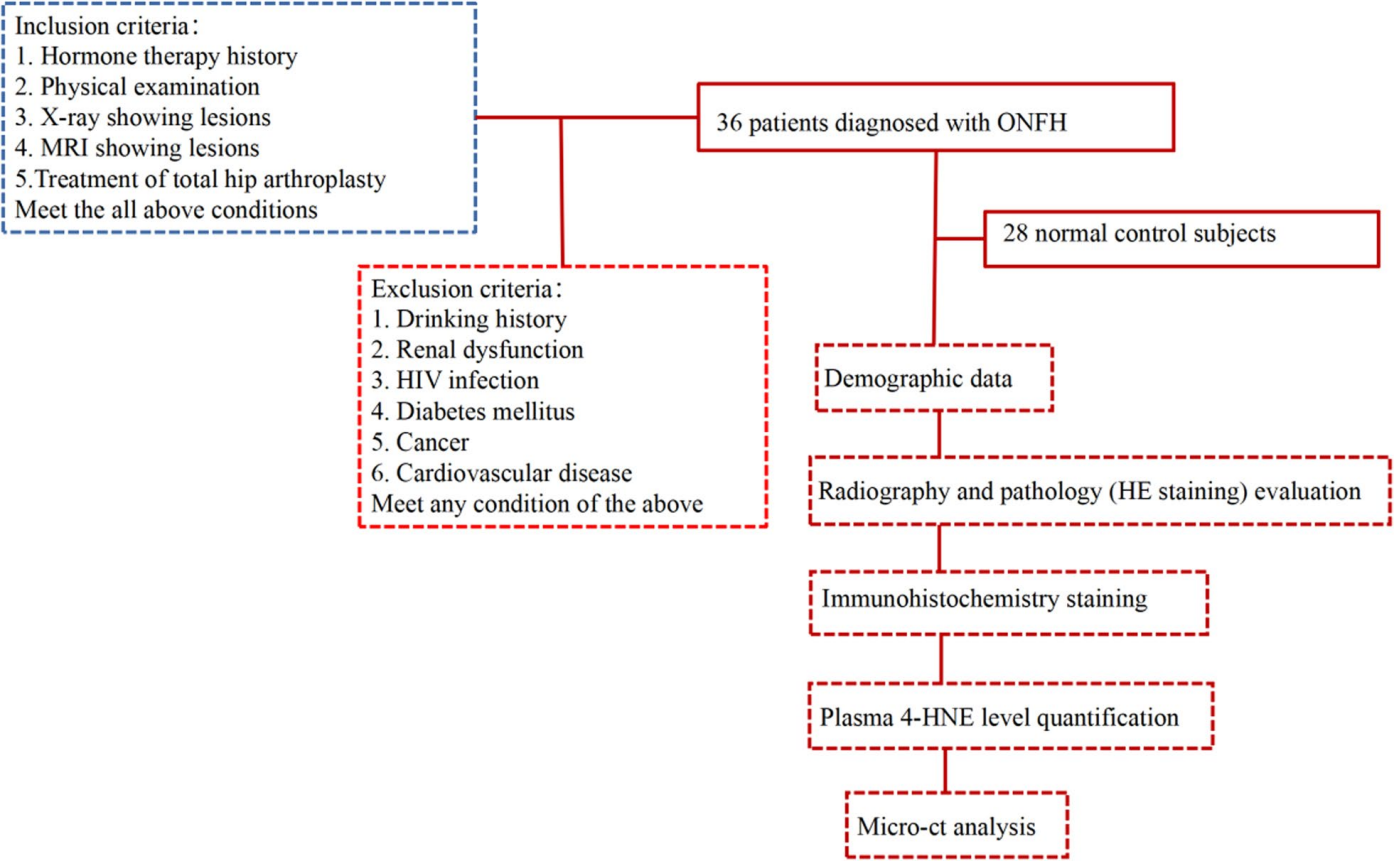
Study flow diagram

This study included 36 SIONFH patients and 28 normal control subjects. We matched the control group participants based on the principle that there were no significant differences between the control group and the disease group at baseline. SIONFH patients from the Third Affiliated Hospital of Guangzhou University of Chinese Medicine who were diagnosed with SIONFH via medical history, physical examinations, X-ray, and MRI between April 2021 and December 2021 were initially included. Patients who were smokers or had renal dysfunction, HIV infection, diabetes mellitus, cancer, and cardiovascular disease were excluded. Normal control subjects were healthy volunteers without hip pain or any lesions shown in anteroposterior and frog-leg lateral pelvic radiographs. According to the Association Research Circulation Osseous (ARCO) staging system (Table 1) [29], the patients were divided into 8 cases of stage II, 19 cases of stage III, and 9 cases of stage IV. Plasma was collected from the patients before arthroplasty. Control subject plasma was obtained from healthy volunteers who received physical examination at the same time. SIONFH bone sections (*n* = 17 total, 3 cases of stage II, 6 cases of stage III, and 8 cases of stage IV) were obtained after THA. As we could not obtain bone tissues from normal individuals, the bone tissue samples of the control group were collected from patients with femoral neck fractures who suffered violent injuries, had no osteoporosis, and had not used antiresorptive drugs (*n* = 6) during the same period. The weight-bearing zone in cartilage tissue was chosen for analysis. Bone samples were collected from healthy tissue or the subchondral necrotic zone at 1–3 mm below the cartilage [12, 30, 31]. The research flowchart is shown in Fig. 1.

**Table 1:** ARCO classification systems of osteonecrosis of the femoral head

Stages	ARCO Classification Systems
I	Normal radiograph, abnormal MRI, or bone scan
II	Abnormal radiograph without fracture or flattening
III	Crescent sign and/or femoral head flattening
IV	Radiographic evidence of arthritis with joint space narrowing, acetabular changes, and/or joint destruction

### Enzyme-linked immunosorbent assay to determine the 4-HNE level

Venous blood samples were obtained from the participants and stored at − 80 °C until the measurement day. Plasma was thawed and tested by sandwich enzyme-linked immunosorbent assay (ELISA) to measure the levels of 4-HNE based on the manufacturer’s protocols (Cusabio, Wuhan, Hubei Province, China).

### Ethical approval

The experiments were undertaken with the understanding and written consent of each subject. The study methodologies conformed to the standards set by the Declaration of Helsinki and were approved by the Ethics Committee of Guangzhou University of Chinese Medicine Third Affiliated Hospital (GYH202101-04).

### H&E staining of bone tissue

Necrotic bone samples, collected from the necrotic area and the femoral head of normal control samples, were fixed in 4% formaldehyde for over 24 h at room temperature. Samples were decalcified using EDTA (10%), dehydrated, and ultimately embedded in paraffin wax. The embedded samples were cut into 5-µm sections, stained using haematoxylin and eosin (H&E), and then observed and captured by a microscope (BX53, Olympus Corp, Japan). Five fields of view were randomly selected under high magnification to count bone lacunae and calculate the percentage of empty lacunae used to indicate the overall apoptosis rate. Percentage of empty lacunae = number of empty lacunae/number of bone lacunae × 100%.

### Immunohistochemistry for 4-HNE

Immunohistochemistry was used to identify 4-HNE protein expression in tissue. Slides were dewaxed and rehydrated with different gradients of xylene and absolute ethanol. H_2_O_2_ (3%) was used to quench endogenous peroxidase activity for 1 h. Antigen retrieval was performed by citrate buffer for 10 min. At room temperature, nonspecific reactivity was blocked in 10% goat serum for 30 min. Then, representative slides were incubated overnight at 4 °C with mouse anti-4-HNE (ab48506, Abcam, Cambridge, MA, USA) antibodies (1:1000 dilution).

After three washes with PBS, the slides were exposed to appropriate secondary biotinylated antibody (goat anti-mouse immunoglobulin (IgG)-horseradish peroxidase HRP; 1:500 dilution; ZSGB-Bio, China) for 30 min at room temperature. Slides were counterstained with haematoxylin, covered with mounting medium, and observed under a microscope (BX53, Olympus).

### Microcomputed tomography analysis

Microcomputed tomography (micro-CT) is the preclinical analogue of clinical CT, providing higher spatial resolution for imaging bone tissue [32, 33]. This enables better observation of microscopic changes in bone tissue. Bone structural parameters mainly included bone volume fraction (BV/TV), trabecular number (Tb. N), trabecular thickness (Tb. Th), and trabecular separation (Tb. Sp). In this study, NEMO Micro CT (NMC-200, China) was used to analyse bone tissue, and a CT analyser program (Avatar 3.0, China) was used to obtain bone structure parameters.

### Post hoc statistical power calculation

Post hoc power analysis provides the critical sample sizes needed to detect statistically significant and clinically meaningful treatment differences and evaluate cost‒benefit ratios so that studies can be conducted with minimal resources without compromising scientific integrity and rigor [34]. Statistical power (1-β) was calculated by online calculators (http://powerandsamplesize.com). The formula based on the obtained data, containing 4-HNE mean levels, standard error, and the number of enrolled patients in different groups, is written below. Statistical power was regarded as strong when the value of statistical power was > 0.8, where the ratio between the sample sizes of the two groups was $\kappa =\frac{{n}_{A}}{{n}_{B}}$. This calculator uses the following formulas to compute sample size and power:

$${n}_{A}=\kappa {n}_{B} \, {\text{and}} \, {n}_{B}=\left(1+\frac{1}{\kappa }\right){\left(\sigma \frac{{z}_{1-\alpha /2}+{z}_{1-\beta }}{{\mu }_{A}-{\mu }_{B}}\right)}^{2}$$

$$1-\beta =\Phi \left(z-{z}_{1-\frac{\alpha }{2}}\right)+\Phi \left(-z-{z}_{1-\frac{\alpha }{2}}\right), z=\frac{{\mu }_{A}-{\mu }_{B}}{\sigma \sqrt{\frac{1}{{n}_{A}}+\frac{1}{{n}_{B}}}}$$

where $\kappa =\frac{{n}_{A}}{{n}_{B}}$ is the matching ratio, σσ is the standard deviation, Φ is the standard normal distribution function, Φ − 1 is the standard normal quantile function, α is type I error, β is type II error, and 1 − β is power.

### Statistical analysis

Data are reported as the mean ± SD (standard deviation) and were analysed using SPSS v23.0 software (SPSS Inc., USA). For comparison between 2 groups, data were analysed by independent t test. For three or more groups, data were examined using ANOVA, followed by the least significant difference test (LSD). Statistical significance was assumed at *P* < 0.05.

## Results

### Demographic data

The average age of the 36 patients (21 males and 15 females) with SIONFH was 39.97 ± 15.23. The average age of the 28 normal control participants (15 males and 13 females) was 42.82 ± 18.33. There were no significant differences in age or sex between groups.

### Radiography and pathology evaluation of SIONFH patients and control subjects

**Fig. 2:**
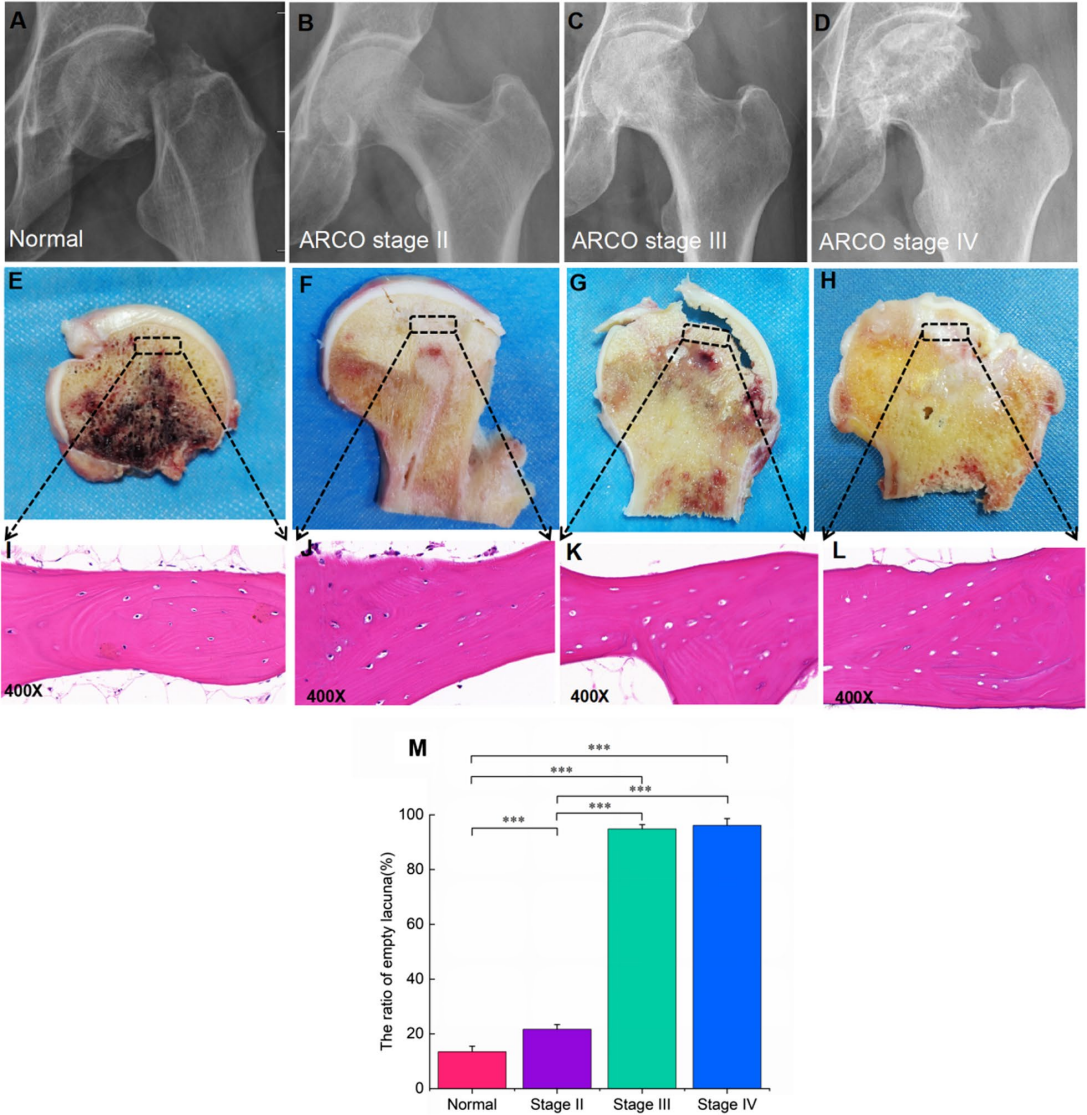
Radiography and pathology result of the control group and SIONFH group.**A–D** X-ray images of control participants and SIONFH patients with different ARCO stages. **E–H** General appearance in the bone and cartilage samples of control participants and SIONFH patients with different ARCO stages. The black dashed boxes indicated the areas collected for further analysis. **I–L** Histopathological features of control and SIONFH bone. **M** The ratio of empty lacunae in SIONFH groups and control group. ***P < 0.001

Radiography results were collected from the normal control subjects and SIONFH patients, representing different SIONFH stages (Fig. 2A–D). Figure 2A presents a homogeneous femoral head density, a normal joint gap, and the position of the fracture line. Figure 2B reveals the nonuniform density and the disappearance of local bone trabeculae. Figure 2C shows a collapsed femoral head with preservation of the joint gap. Figure 2D demonstrates a deformed femoral head, narrow joint space, and joint destruction. Figure 2E–H shows the general appearance of femoral head sections. Figure 2E reveals a normal femoral head from a femoral neck fracture patient. Figure 2F shows the disorderly bone trabeculae in the necrotic part and the smooth joint surface. Figure 2G presents a distinct collapsed femoral head, rough cartilage surface, and more disorganized bone trabeculae. Figure 2H shows the cartilage structure thinning, degeneration, and severe cartilage folds. Figure 2I–L shows the HE staining results. Figure 2I shows an intact bone structure, and the osteophytes fill the lacunae. Figure 2J–L presents gradually increasing empty lacunae in necrotic osteocyte bone trabeculae based on increased stage, indicating substantial bone cell loss. Furthermore, Fig. 2M shows that the ratio of empty lacunae in the normal control group was lower than that in the SIONFH group (*P* < 0.001). The ratio of empty lacunae in stages III and IV was higher than that in stage II (*P* < 0.001). There were no significant differences between stage III and stage IV (*P* > 0.05).

### Immunohistochemical staining of SIONFH patients and control subjects

**Fig. 3:**
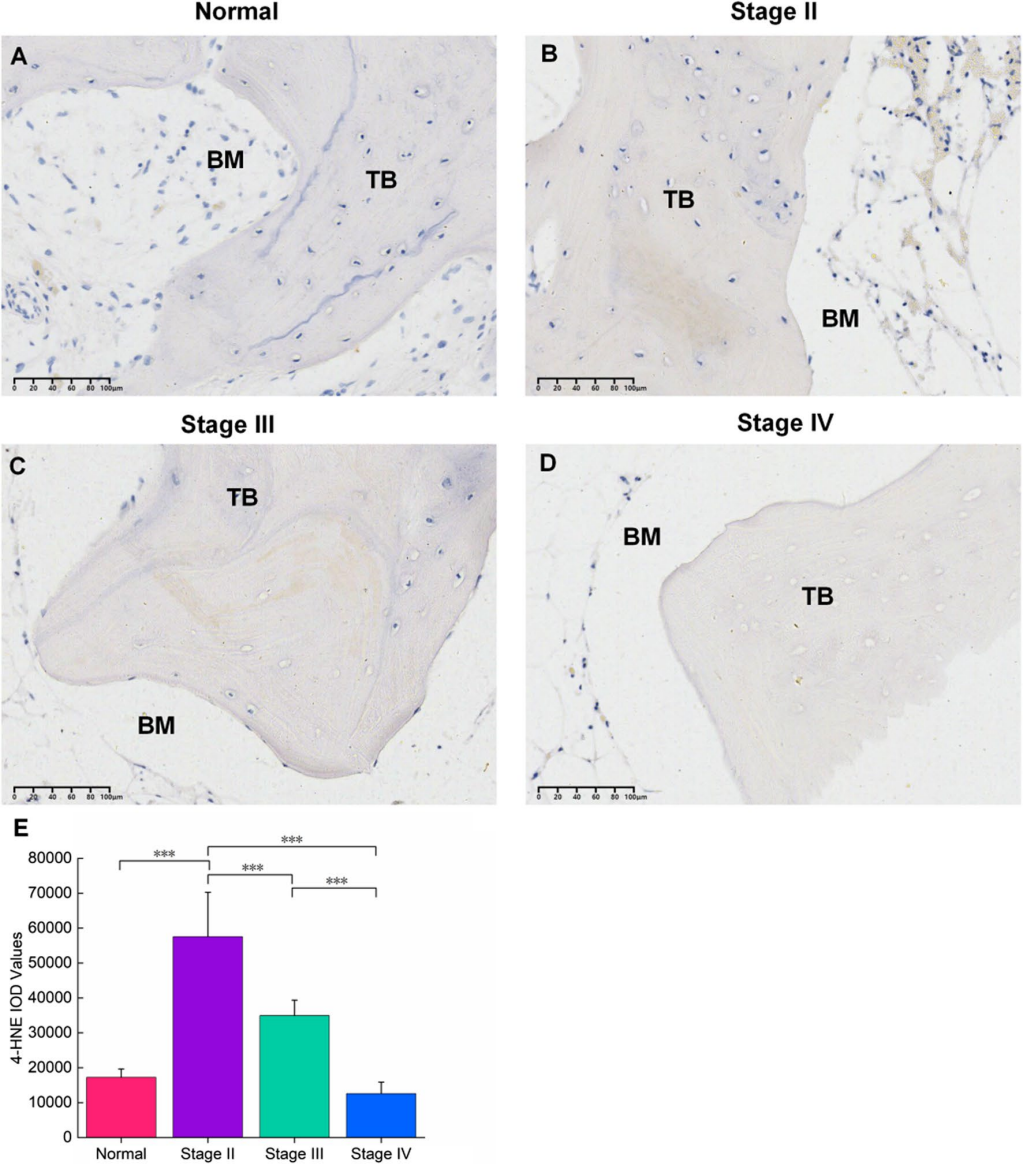
Immunohistochemistry results for 4-HNE of bone samples in the control and SIONFH groups with different stages. **A** A low-level presence of 4-HNE was observed in healthy samples. **B–D** The levels of 4-HNE decreased as the ARCO stage progressed. **E** The IOD value of immunohistochemistry for 4-HNE. ***P < 0.001. TB: trabecular bone; BM: bone marrow

Immunohistochemical staining was used to assess 4-HNE expression in the necrotic area and normal control tissues (Fig. 3A-D). 4-HNE was determined to be positive on trabecular bone, osteocytes, and bone marrow. Low expression of 4-HNE was detected in healthy tissue, whereas high expression was observed in stage II to III necrotic bone. 4-HNE is negatively associated with the progression of SIONFH.

### Plasma 4-HNE level quantification in SIONFH patients and control subjects

**Fig. 4:**
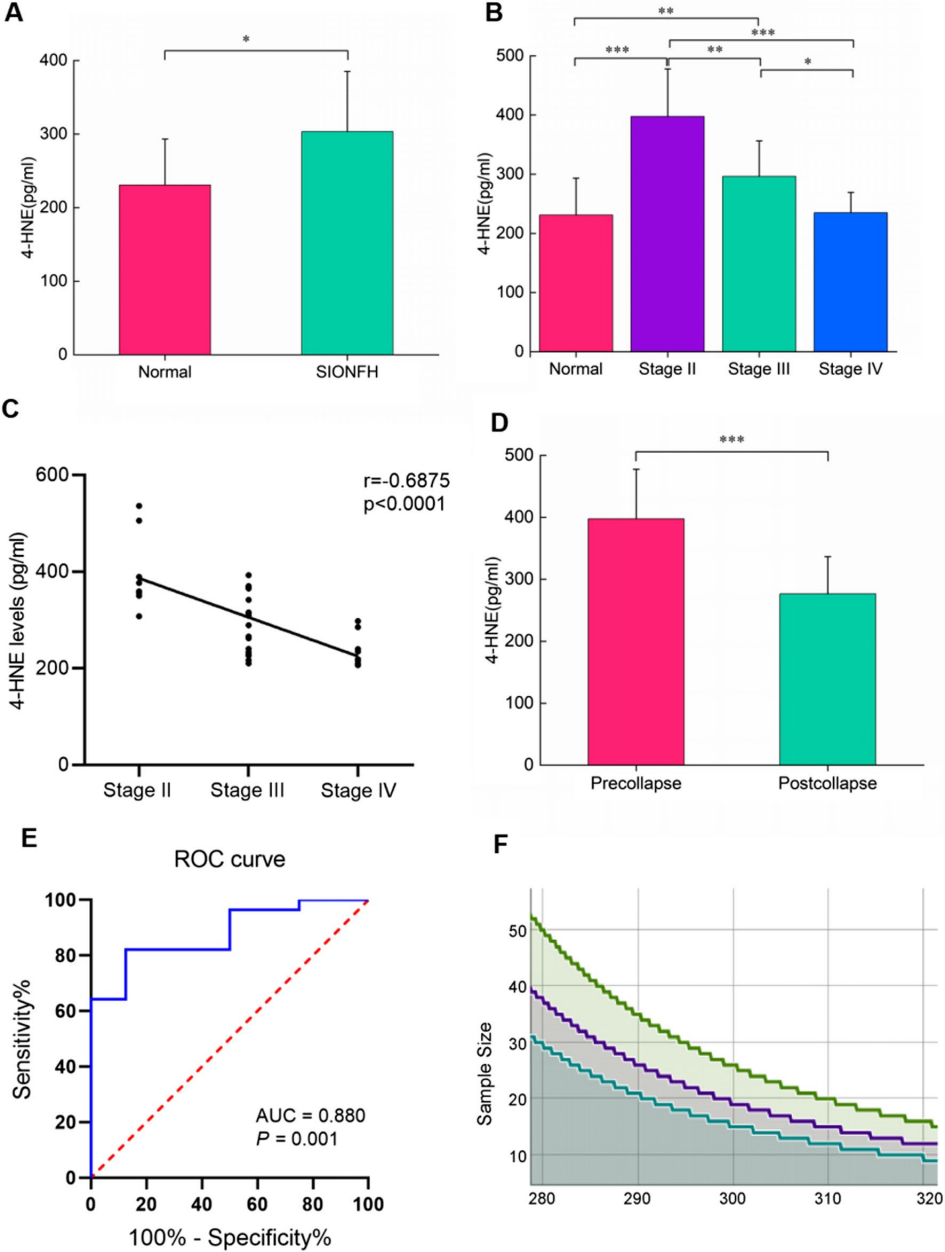
The expression level comparison of 4-HNE in patients with femoral head necrosis at different stages and control group and relevance assessment. **A** 4-HNE levels of the SIONFH group were significantly higher than that of the control group. **B** 4-HNE levels with statistical differences among different groups. **C** Graph showing the negative correlation between 4-HNE levels and ARCO stages. **D** 4-HNE levels were decreased in the postcollapse group. **E** The receiver operating characteristic (ROC) curve and the area under the curve (AUC) are associated with the sensitivity and specificity of SIONFH. **F** Statistic power is determined by mean and sample size. Statistic power: green line for 0.9, purple line for 0.8, and blue line for 0.7. ***P < 0.001

Table 2 shows the plasma 4-HNE levels determined by ELISA. The plasma 4-HNE levels were higher in patients with SIONFH than in healthy participants (*P* < 0.001) (Fig. 4A). Moreover, plasma 4-HNE levels varied significantly among the different ARCO stages (Fig. 4B). The levels of 4-HNE were 397.64 ± 80.19 pg/ml, 296.18 ± 60.21 pg/ml, and 235.02 ± 34.15 pg/ml in stage II, stage III, and stage IV, respectively. Furthermore, a negative correlation was observed between the 4-HNE level and ARCO stage (*r* =  − 0.6875, *P* < 0.001) (Fig. 4C). The 4-HNE level of the precollapse group was noticeably higher than that of the postcollapse group (*P* < 0.001) (Fig. 4D). The area under the curve (AUC), which was calculated by ROC curve analysis to determine the values for 4-HNE levels in the SIONFH group compared with those in the control group, was 0.744 (*P* < *0.001*). The sensitivity was 87.5%, and specificity was 82.1% (cut-off, 345.81 pg/ml) (Fig. 4E). After calculation, the statistical power was 0.9445, suggesting that the sample size of 36 and the sampling ratio of 0.56 were sufficient to obtain the conclusion (Fig. 4F).

**Table 2:** Plasma 4-HNE levels in SIONFH patients and control group and potential relation between other clinical data

Group	Cases	4-HNE level (pg/mL)	Comparison	*P* value
Control	28	230.89 ± 62.29	Control vs SIONFH	< 0.001
SIONFH	36	303.44 ± 81.72		
ARCO stages				
Stage II	8	397.64 ± 80.19	II vs III	0.001
Stage III	19	296.18 ± 60.21	III vs IV	0.031
Stage IV	9	235.02 ± 34.15	II vs IV	< 0.001
Precollapse	8	397.64 ± 80.19	Pre- vs Post-	< 0.001
Postcollapse	28	275.52 ± 60.07

### Micro-CT analyses of SIONFH patients and control subjects

**Fig. 5:**
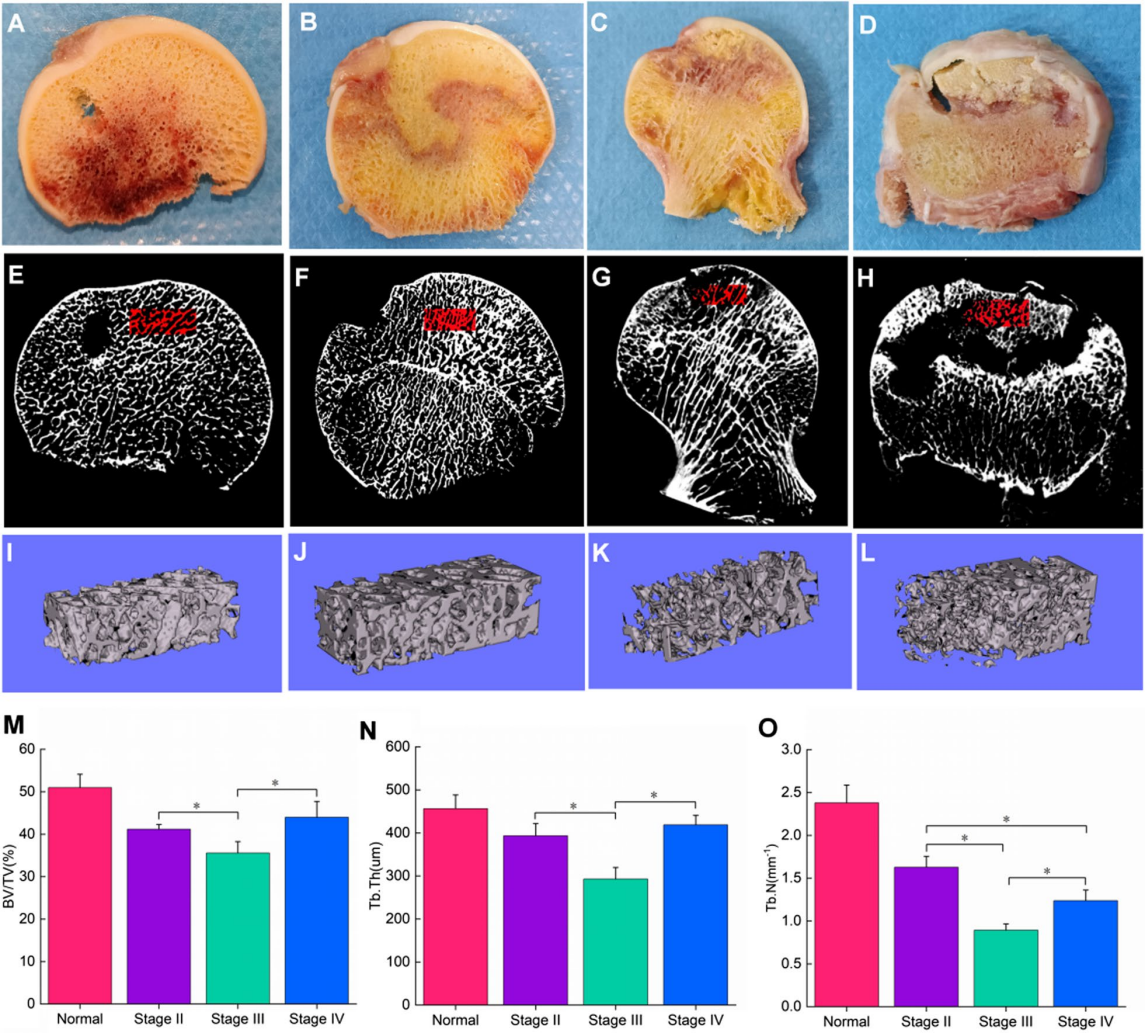
The structural characteristics of bone in SIONFH patients and control subjects. **A–D** General images of bone samples of a control subject and SIONFH patients with different ARCO stages. **E–H** Micro-CT two-dimensional images of bone samples from the control subject and SIONFH patients with three ARCO stages. **I–L** Three-dimensional micro-CT images of ROI in bone samples from control and SIONFH bone samples. **M–O** Decreased value of BV/TV, Tb. Th and Tb. N in stages II and III. Higher values of BV/TV, Tb. N, Tb. Th in stage IV than that in stage III was observed. *P < 0.05

The micro-CT results showed that the BV/TV, Tb. Th and Tb. N of SIONFH patients were lower than those of the normal group. Moreover, the structure and number of trabecular bones in the necrotic area differed among different stages (Fig. 5A–H). The results showed that BV/TV, Tb. Th and Tb. N decreased in stages II and III but slightly increased in stage IV. This means that the structural characteristics of bone in stage IV are significantly changed (Fig. 5M–O).

## Discussion

The overall purpose of this cross-sectional study was to assess the relationship between plasma 4-HNE levels and the progression of SIONFH. The plasma 4-HNE levels were higher in the in SIONFH patients than in normal control subjects. Moreover, 4-HNE levels decreased with advancing ARCO stage. The histopathology and radiography revealed the destruction of the bone structure was also gradually aggravated along with the progression of SIONFH. Therefore, this study indicates that 4-HNE expression may be associated with collapse of the femoral head. To our knowledge, this is the first study to demonstrate the association between 4-HNE expression and the disease severity of SIONFH.

SIONFH is the most common type of nontraumatic femoral head necrosis and is easily overlooked in patients with prolonged steroid use. A study involving 3000 nontraumatic osteonecrosis patients in 1998 found that corticosteroids were engaged in over 33% of cases [35]. In 2011, a prospective study containing 1199 hip and knee samples from 302 participants who received hormonal therapy for at least one year reported that the incidence of osteonecrosis was 37% in patients with systemic lupus erythematosus (SLE) but only 21% in patients without SLE [36]. However, the pathogenesis of SIONFH remains to be elucidated at the molecular level. The ultimate mechanism is compromised blood flow with failure to deliver nutrients to the affected bone, favoured by numerous preexisting factors [37]. This study suggests that 4-HNE may be related to the progression of SIONFH.

4-HNE is a common marker of oxidative stress [21, 22]. Researchers have conducted in-depth research on the existence of 4-HNE and its possible pathogenesis in the nervous, cardiovascular, respiratory, and urinary systems in the past ten years [38]. Wataya et al. found that 4-HNE is a physiological constituent of various tissues [39]. Tsuneyama et al. suggested that HNE is a marker of liver injury and one of the pathogenic factors in liver cirrhosis [40]. Butterfield et al. concluded that HNE may be a crucial factor in Alzheimer’s disease [41]. Moreover, the key to oxidative stress is mitochondrial damage, which releases many reactive oxygen species, destroys cell redox balance, and regulates cell fate [42]. It has been found that oxidative stress caused by long-term use of glucocorticoids (GCs) can impair the repair ability of bone tissue [43]. In this study, the results from radiography, general view of the femoral head sample, and HE staining demonstrated that the pathological features of SIONFH are aggravated osteocyte oxidative stress and an increasing number of lacunae as SIONFH progresses. The ELISA results showed that the 4-HNE level in the SIONFH group was higher than that in the normal control group. The 4-HNE level decreases with the progression of SIONFH. Furthermore, we found that 4-HNE is associated with ARCO stages by relevant analysis. This indirectly suggests that decreased 4-HNE levels may be used as a marker to predict the collapse of SIONFH and the severity of bone cell oxidative stress. Furthermore, it may be that the high expression of 4-HNE in the early stage of SIONFH (stage II) aggravates the oxidative stress of osteocytes, which may disrupt the balance between osteoblast and osteoclast activities, leading to the progression of femoral head collapse. However, the decreased 4-HNE level after collapse (stage III and stage IV) may be a bad signal that may be associated with nonvital bone with a large number of empty osteocyte lacunae. Therefore, the cut-off value calculated from the ROC curves can help to determine a specific collapse concentration.

Oxidative stress is closely related to bone homeostasis. Previous studies have found that excessive oxidative stress inhibits osteoblast proliferation, and intervention with antioxidants can effectively protect and restore their osteogenic function [44]. Other studies also found that when osteoblasts are exposed to dexamethasone, intracellular ROS levels increase and cause cell dysfunction by inhibiting the expression of several osteogenic markers, including alkaline phosphatase, runt-related transcription factor 2, bone morphogenetic protein, osteonectin, and osteocalcin [45]. Moreover, the hyperactivity of osteoclasts is another important pathological mechanism of SIONFH [19]. ROS can control the differentiation and maturation of osteoclasts and promote osteoclast proliferation [46]. The micro-CT results showed that bone histomorphometry parameters (BV/TV, TB. N, and Tb. Th) in SIONFH patients were lower than those in normal controls. The bone histological parameters of stage III patients were lower than those of stage II patients, which may be related to the fracture of the necrotic area of the femoral head in stage III patients. The bone histological parameters of stage IV patients were improved compared with those of stage II and III patients, which may be closely related to the development of osteoarthritis in stage IV SIONFH patients.

4-HNE is a small and highly reactive molecule in the process of oxidative stress, which may link genomics and proteomics. The study of HNE as a bioactive marker of SIONFH may open a new research direction in this field. 4-HNE may be applied to modify pathophysiological processes, and the use of substances that act against 4-HNE may be developed into advanced adjuvant therapies. These are certainly the future directions of 4-HNE research.

There are some limitations in our study. First, we did not evaluate 4-HNE levels in stage I patients due to the difficulty of enrolling patients at an early stage of SIONFH. Second, the small sample size limited the accuracy of the research. Even with these limitations, our study is the first to demonstrate that 4-HNE could be used as a marker for the progression of SIONFH.

## Conclusion

This study primarily identifies lower levels of 4-HNE as a critical biomarker for the progression of SIONFH. The 4-HNE level is elevated in ARCO stage II patients and subsequently decreases with the progression of ARCO staging. The cut-off concentration (345.81 pg/ml) of 4-HNE may remind clinicians to monitor the disease progression of SIONFH to avoid collapse closely. Using substances acting on 4-HNE for experimental research represents a promising direction for our future research.

## Data Availability

The data supporting this study’s findings are available from the corresponding author, QW, upon reasonable request.
